# Zinc Oxide Nanoparticles Alleviate Salt Stress in Cotton (*Gossypium hirsutum* L.) by Adjusting Na^+^/K^+^ Ratio and Antioxidative Ability

**DOI:** 10.3390/life14050595

**Published:** 2024-05-07

**Authors:** Jiajie Qian, Ren Shan, Yiqi Shi, Huazu Li, Longshuo Xue, Yue Song, Tianlun Zhao, Shuijin Zhu, Jinhong Chen, Meng Jiang

**Affiliations:** 1 Hainan Institute, Zhejiang University, Yazhou Bay Science and Technology City, Sanya 572025, China; 22216131@zju.edu.cn (J.Q.); 22316121@zju.edu.cn (R.S.); 22116141@zju.edu.cn (Y.S.); yuesong@zju.edu.cn (Y.S.); tlzhao@zju.edu.cn (T.Z.); shjzhu@zju.edu.cn (S.Z.); 2 College of Agricultural and Biotechnology, Zhejiang University, Hangzhou 310058, China; 12016016@zju.edu.cn (H.L.); xuelongshuo@163.com (L.X.)

**Keywords:** antioxidative system, cotton, Na/K ratio, soil salinization, ZnO NPs

## Abstract

Soil salinization poses a threat to the sustainability of agricultural production and has become a global issue. Cotton is an important cash crop and plays an important role in economic development. Salt stress has been harming the yield and quality of many crops, including cotton, for many years. In recent years, soil salinization has been increasing. It is crucial to study the mechanism of cotton salt tolerance and explore diversified materials and methods to alleviate the salt stress of cotton for the development of the cotton industry. Nanoparticles (NPs) are an effective means to alleviate salt stress. In this study, zinc oxide NPs (ZnO NPs) were sprayed on cotton leaves with the aim of investigating the intrinsic mechanism of NPs to alleviate salt stress in cotton. The results show that the foliar spraying of ZnO NPs significantly alleviated the negative effects of salt stress on hydroponic cotton seedlings, including the improvement of above-ground and root dry and fresh weight, leaf area, seedling height, and stem diameter. In addition, ZnO NPs can significantly improve the salt-induced oxidative stress by reducing the levels of MDA, H_2_O_2_, and O_2_^−^ and increasing the activities of major antioxidant enzymes, such as superoxide dismutase (SOD), peroxidase (POD), and catalase (CAT). Furthermore, RNA-seq showed that the foliar spraying of ZnO NPs could induce the expressions of *CNGC*, *NHX2*, *AHA3*, *HAK17*, and other genes, and reduce the expression of *SKOR*, combined with the *CBL-CIPK* pathway, which alleviated the toxic effect of excessive Na^+^ and reduced the loss of excessive K^+^ so that the Na^+^/K^+^ ratio was stabilized. In summary, our results indicate that the foliar application of ZnO NPs can alleviate high salt stress in cotton by adjusting the Na^+^/K^+^ ratio and regulating antioxidative ability. This provides a new strategy for alleviating the salt stress of cotton and other crops, which is conducive to the development of agriculture.

## 1. Introduction

Recently, the majority of agricultural land has experienced abiotic stress, which can significantly decrease crop yields [1]. Soil salinity stress is a common type of abiotic stress, a prevalent issue in arid and semi-arid regions, and arises from the accumulation of salts in the soil, adversely impacting crop growth and agricultural productivity [2]. High salt concentrations in the soil restrict water and nutrient uptake by plants, leading to osmotic stress and nutritional deficiencies for essential physiological processes [3]. Furthermore, the presence of excess salts disrupts the ion balance in plants, causing ion toxicity and metabolic imbalances, resulting in the efflux of K^+^ and Ca^2+^, and ultimately leading to the dehydration of the plant [4]. Additionally, these contribute to the over-accumulation of reactive oxygen species (ROS) in plants, causing oxidative damage to cellular components, including lipids, proteins, and DNA, and disrupting key plant biological activities, thus further exacerbating the adverse effects [5,6]. Therefore, addressing this challenge is crucial for ensuring the resilience of agricultural systems and environmental health.

Crops resist high-salt-stress environments through multiple mechanisms. Regulating ion homeostasis, alleviating osmotic stress, regulating cytoskeletal dynamics, optimizing cell wall composition, and mediating plant hormone signal transduction are common pathways [7]. For example, relevant studies have shown that PIP3 regulates salt tolerance in Arabidopsis by establishing a novel ligand–receptor signaling cascade through RLK7 [8]; plant hormones, such as gibberellin acid (GA) and salicylic acid (SA), can improve the growth and development of cotton plants under salt stress by regulating the expression levels of related genes [9]; functional protein OsASR6 in rice plants under salt stress activates the antioxidant system and reestablishes cellular ion homeostasis [10].

Cotton (*Gossypium hirsutum* L.) is one of the most important fiber crops, growing well in tropical and subtropical regions, and accounting for about 35% of the total fiber in the world [11]. Cotton faces a variety of biotic and abiotic stresses during its life cycle, among which salinization has emerged as one of the major threats to sustainable cotton production worldwide [12]. In order to better adapt to salt stress environments, cotton plants have developed various physiological and biochemical mechanisms, mainly including ion transport and uptake, the biosynthesis of osmoprotectants and compatible solutes, the activation of antioxidant enzymes and the synthesis of antioxidants, the synthesis of polyamines, and hormonal regulation [13]. Cotton has also evolved several signaling pathways to cope with highly saline soil, for example, ABA is involved in plant development, reproduction, and regulation of the stress response, and transcription factors that regulate the expression of downstream genes are involved in suppressing salt stress in plants [14].

Nanotechnology now embodies great potential and broad application prospects for sustainable and efficient agricultural productivity and crop yields as well as improved food security [15,16,17]. Nanoparticles can be better absorbed and utilized by plants due to their small particle size. Currently, nanoparticles are used in agriculture due to their unique physical and chemical properties and are used in various aspects of the agricultural industry, such as nano-biofertilizers, the packaging of agricultural products for processing, and the detection of food contaminants [18,19,20]. In particular, nanomaterials can mimic the action of antioxidant enzymes, such as superoxide dismutase (SOD), peroxidase (POD), and catalase (CAT), to alleviate the adverse growth of plants under abiotic stresses [21]. Zinc oxide nanoparticles (ZnO NPs) are one of the most widely used nanoparticles in the field of nanotechnology and widely used in the pharmaceutical, medical, cosmetic, food, agricultural, and biomedical fields due to their unique physical and chemical properties [22]. In the field of agriculture, ZnO NPs are used in various applications, such as nano-fertilizers, nano-growth regulators, and nano-pesticides [23]. ZnO NPs can be used as a new agricultural antiviral material as a synergistic nutrient supply and immune regulation means to control virus-infected crops [24]. Under different stress conditions, ZnO NPs increase photosynthetic pigments, decrease antioxidant enzymes, and accumulate non-enzymatic antioxidants in response to stress. It has been shown that the foliar spraying of maize and rice with ZnO NPs minimized oxidative stress and increased enzyme activities to alleviate cadmium (Cd) stress on crops [25,26]. It has been shown that the foliar spraying of ZnO NPs can promote the growth of tomato, sorghum, and barley and effectively alleviate salt stress [27,28,29].

Previous studies have shown that the foliar application of nano-zinc improved the growth parameters and yield of cotton under salt stress conditions, and cerium oxide nanoparticles could enable a better ability to maintain the cytosolic K^+^/Na^+^ ratio to improve cotton salt tolerance [30,31]. Meanwhile, the effect of ZnO NPs in mitigating salt stress in cotton is still unclear. Therefore, our aim was to observe the effects of ZnO NPs on alleviating salt stress in this study, explore the internal mechanism of ZnO NPs in improving salt tolerance of cotton seedlings, and provide a new scheme for alleviating crop salt stress. Eventually, we found that in hydroponically grown cotton seedlings, the foliar application of ZnO NPs could mitigate the salt stress by fine-tuning the Na^+^/K^+^ ratio and regulating their antioxidative ability. It is worth noting that since zinc is a heavy metal element, the effects of spraying ZnO NPs on environmental pollution and food safety need to be further evaluated and controlled.

## 2. Materials and Methods

### 2.1. Cotton Materials and Treatments

The cotton (*Gossypium hirsutum* L.) seeds used were evenly plump and of the same size. First, they were immersed in purified water at 25 °C for one night. After absorbing the moisture on the seed surface, the seeds were sowed in a 50-hole seedling tray (with a ratio of nutrient soil to vermiculite of 1:1). The procedure of germination and growth was at 25 °C and 70% humidity. After seed germination, multiple misting sprays of water facilitated shell shedding and cotyledon spreading. After the two cotyledons of the cotton seedlings were completely unfolded and flattened, the seedlings with consistent growth were selected and transferred to 1 L black perforated plastic buckets (care was taken to avoid damage to the root system during the transfer process), which were fixed using 25 mm-diameter planting cotton and cultivated using modified Hoagland nutrient solution, which was replaced every 3 days.

The NPs of ZnO were purchased from Chaowei Nano Technology Co., Ltd. (Shanghai, China). The ZnO NPs were spherical with regular diameters of 30 nm. To minimize agglomeration, we sonicated the different concentrations of ZnO NP suspensions for 1 h before use. The leaves were sprayed with 10 mL of water and ZnO NP solutions of 50, 100, 150, and 200 mg/L once a day, evenly on the leaf surface and back, for seven days. On the day of salt treatment, 150 mM of NaCl was added to the nutrient solution of the hydroponic box, while the control group was untreated. Samples were taken at 24 h and 6 days after the salt treatment for later measurement.

### 2.2. Determination of Agronomic Traits

Cotton plants were randomly chosen from the salt treatment after six days of treatment. The morphological characteristics of the cotton plants, such as the seedling height, fresh weight of root and above-ground parts, and stem diameter were determined. The root and above-ground parts were dried at 60 °C until a constant weight, and the dry weight was recorded. A leaf area analyzer was used to determine the leaf area of cotton seedlings, and the third true leaf of each group of plants was selected for the determination.

### 2.3. Determination of Chlorophyll Content and Chlorophyll Fluorescence Parameters

The chlorophyll content of the samples salt-treated for 6 d was measured using a convenient chlorophyll meter (SPAD-502 Plus), with three points averaged to obtain one value per true leaf. A PEA was used to measure the chlorophyll fluorescence parameters after 30 min of dark adaptation. Well-grown, uniform, healthy, and disease-free cotton seedlings were selected for the measurement, and the functional leaves were the 3rd true leaves of the cotton seedlings.

### 2.4. Determination of Elements

The nitrogen (N) elemental content was determined by the fully automatic Kjeldahl nitrogen method. The phosphorus (P), potassium (K), sodium (Na), zinc (Zn), calcium (Ca), and sulfur (S) elemental contents were determined by ICP-AES. The functional leaves were the 3rd true leaves of the cotton seedlings.

### 2.5. Measurement of Antioxidant Enzyme Activities and MDA, H_2_O_2_, O_2_^−^, Pro, Protein, and AA Contents

The samples were sampled for 6 d of salt treatment, and the activities of SOD, POD, and CAT, as well as the contents of malondialdehyde (MDA), superoxide anion (O_2_^−^), hydrogen peroxide (H_2_O_2_), proline (Pro), soluble proteins, and amino acid (AA) in the cotton leaves were determined by the kit method. We took 0.1 g of leaf tissue and added 1 mL of extraction solution, homogenized it in an ice bath, centrifuged it at 8000/10,000 rpm and 4 °C for 10/20 min, and took the supernatant as the solution to be measured. We preheated the enzyme counter for more than 30 min, added the test solution and the reaction reagent sequentially into a 96-well plate, and measured the absorbance value at the corresponding wavelength.

### 2.6. Transcriptome Sequencing (RNA-Seq)

When the cotton seedlings entered the two-leaf-one-heart stage, plants with uniform growth were selected and sprayed with 100 mg/L ZnO NPs solution for 7 days. After spraying, a 150 mM salt-stress treatment was applied. The sampling time point was 24 h after the salt-stress treatment, the sampling site was the third true leaf of cotton, and three independent biological replicates were obtained for each treatment. We selected DESeq2-identified genes with adjusted an *p*-value (Padj) < 0.05 as differentially expressed genes [32].

### 2.7. Statistical Analyses

Preliminary calculations were made using Microsoft Excel to organize the experimental data; one-way ANOVA was performed using SPSS 29.0; multiple comparisons among treatments and significance difference tests were performed using Duncan’s method (*p* < 0.05), and graphing was performed using Prism 10 software.

## 3. Results

### 3.1. Effects of ZnO NPs on Phenotypic Characteristics of Cotton Seedlings under Salt Stress

**Figure 1:**
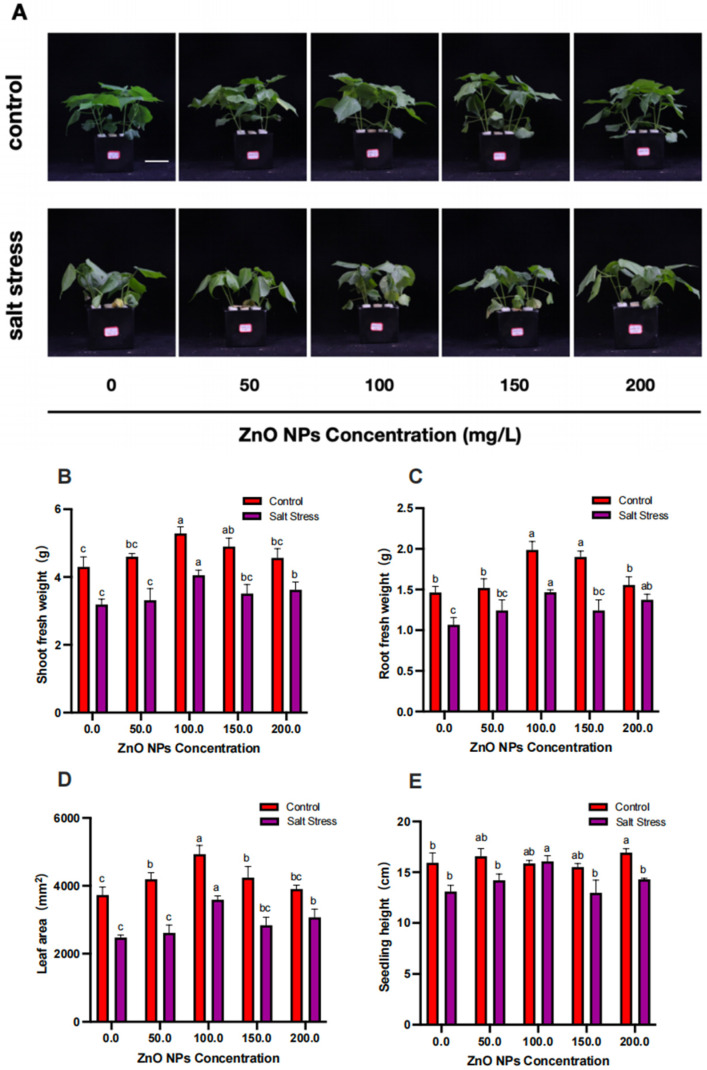
ZnO NPs alleviate salt stress in cotton. The phenotypes (**A**), shoot fresh weights (**B**), root fresh weights (**C**), leaf areas (**D**), and seedling heights (**E**) of cotton seedlings under salt stress and subjected to 0, 50, 100, 150, and 200 mg/L of ZnO NPs. Scale bar = 100 mm. The data given are the averages of three replicates, with the standard deviation (SD) shown by the error bars. Different letters above or below the error bars show the differences at *p* < 0.05.

To evaluate the effect of ZnO NP application on salt stress, after the third true leaf of the cotton plant had grown, 150 mM NaCl was added to the nutrient solution to simulate the salt-stressed environment. After 6 days of salt-stress treatment, the shoot fresh weight, root fresh weight, leaf area, and seeding height significantly decreased (*p* < 0.05) by 25.8%, 27.1%, 33.6%, and 17.8%, respectively, compared to those of the untreated control (Figure 1B–E). The shoot dry weight, root dry weight, and stem diameter significantly decreased (*p* < 0.05) by 17.1%, 15.3%, and 16.1% after the salt-stress treatment (Figure S1) compared to the untreated control.

After the foliar application of 50, 100, 150, and 200 mg/L ZnO NPs, we found that the growth inhibition of cotton under salt stress was significantly alleviated, as evidenced by the fact that the cotton seedlings under salt stress maintained better agronomic traits than the control. We also measured after 6 days of salt treatment, and the results suggest that spraying 100 mg/L ZnO NPs was the most effective in alleviating high-salinity toxicity in cotton plants, with all indices significantly induced (*p* < 0.05) from 22.67% to 50.68% (Figure 1B–E and Figure S1).

### 3.2. Effects of ZnO NPs on Chlorophyll Content and Chlorophyll Fluorescence Parameters of Cotton Seedlings under Salt Stress

**Figure 2:**
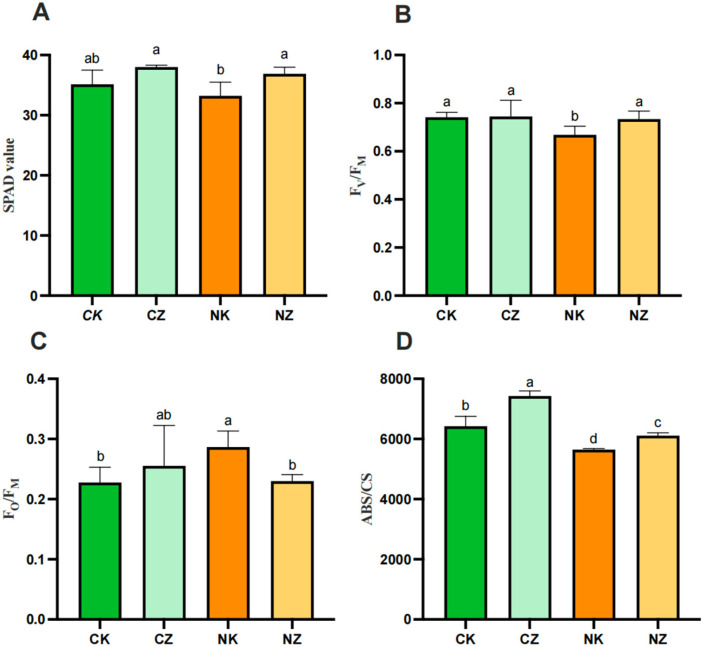
Effects of ZnO NPs on chlorophyll content and chlorophyll fluorescence parameters of cotton seedlings under salt stress. (**A**) SPAD value; (**B**) F_V_/F_M_, maximum photochemical efficiency, which can be used as a reliable index of photochemical activity of photosynthetic apparatus; (**C**) F_O_/F_M_, quantum ratio for heat dissipation; (**D**) ABS/CS, light energy absorbed per unit leaf area. Control (CK), CK treated with 100 mg/L ZnO NPs (CZ), CK treated with 150 mM NaCl (NK), and CZ treated with 100 mg/L ZnO NPs (NZ). The data given are the averages of three replicates, with the standard deviation (SD) shown by the error bars. Different letters above or below the error bars show the differences at *p* < 0.05.

The responses of the SPAD, F_V_/F_M_, F_O_/F_M_, and ABS/CS of cotton seedlings under salt stress to ZnO-NPs are illustrated in Figure 2. Compared to the CK, salt stress significantly reduced (*p* < 0.05) the SPAD, F_V_/F_M_, and ABS/CS of the cotton seedlings by 5.44%, 9.97%, and 12.14%, respectively. Inversely, the SPAD, F_V_/F_M_, and ABS/CS were remarkably increased (*p* < 0.05) by ZnO-NPs. The difference was that the F_O_/F_M_ was significantly higher (*p* < 0.05), by 25.84%, in the plants under salt stress compared to the CK, but decreased significantly (*p* < 0.05) after the application of ZnO NPs, basically returning to the level of the CK. A comparison with the plants under non-salt stress revealed that only the ABS/CS was significantly increased (*p* < 0.05), by 15.63%, after spraying with ZnO NPs, and there were no significant differences in the SPAD, F_V_/F_M,_ or F_O_/F_M_.

### 3.3. Effects of ZnO NPs on Elemental Contents of Cotton Seedlings under Salt Stress

**Figure 3:**
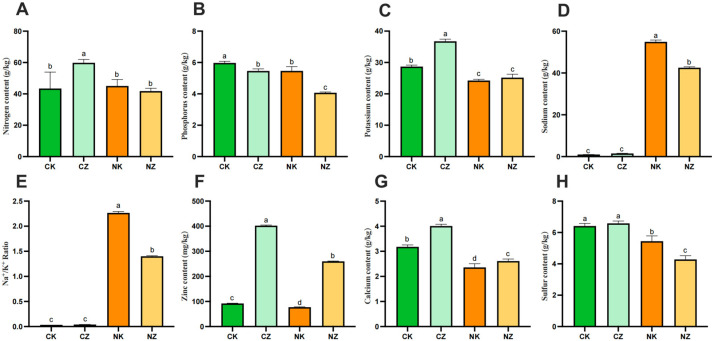
Effects of ZnO NPs on elemental contents of cotton seedlings under salt stress. (**A**) Nitrogen content; (**B**) phosphorus content; (**C**) potassium content; (**D**) sodium content; (**E**) Na^+^/K^+^ Ratio; (**F**) zinc content; (**G**) calcium content; (**H**) sulfur content. Control (CK), CK treated with 100 mg/L ZnO NPs (CZ), CK treated with 150 mM NaCl (NK), and CZ treated with 100 mg/L ZnO NPs (NZ). The data given are the averages of three replicates, with the standard deviation (SD) shown by the error bars. Different letters above or below the error bars show the differences at *p* < 0.05.

Mineral elements play a crucial part in the regulation of plant growth and development. N, P, and K are indispensable nutrients for plant growth and development, Zn plays an important role in photosynthesis, Ca affects osmotic regulation, and S affects metabolism. The results of the determination of the N, P, K, Na, Zn, Ca and S contents of the whole plants of cotton seedlings treated with 100 mg/L of ZnO NPs are shown in Figure 3. In terms of the N, P and K contents (Figure 3A–C), under no stress condition, the N and K contents after spraying ZnO NPs were significantly higher than that of the CK, while the P content was significantly lower than that of the CK. Under 150 mM of salt stress, the K content after spraying ZnO NPs was a little higher but not significantly different from that of the CK, while the P content was significantly lower than that of the CK. Under salt stress, the internal sodium content was significantly higher than that of the CK and significantly decreased after spraying with ZnO NPs (Figure 3D), as was the Na^+^/K^+^ ratio (Figure 3E). The Zn contents in the leaves of the cotton seedlings in the CZ and NZ groups were significantly higher due to the foliar spraying with ZnO NPs (Figure 3F). Salt stress also significantly reduced the contents of elemental Ca and S, which were significantly higher and lower, respectively, after spraying with ZnO NPs (Figure 3G,H).

### 3.4. Spraying of ZnO NPs Alleviated Salt Stress-Induced Oxidative Stress

**Figure 4:**
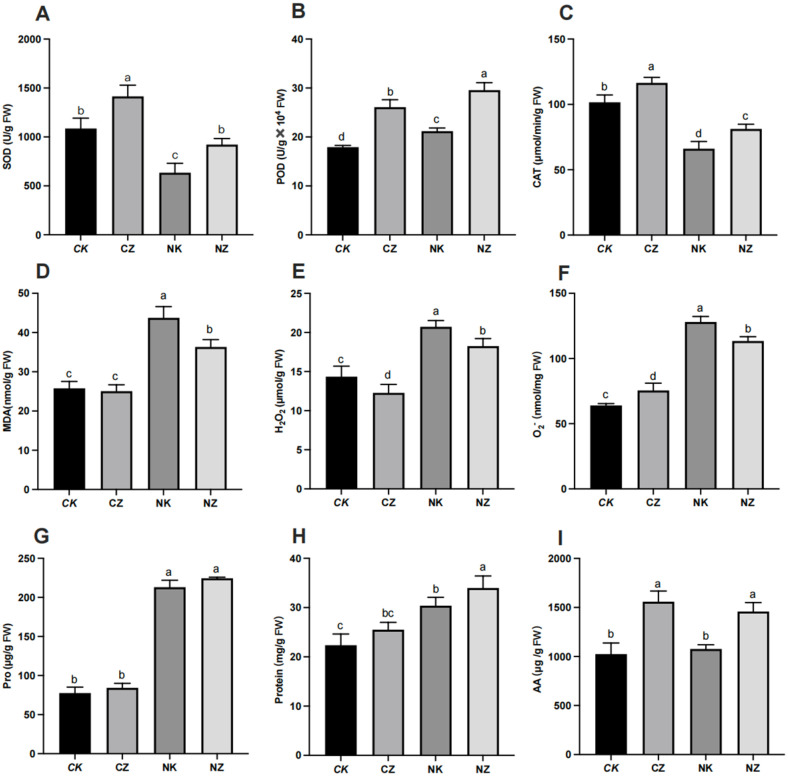
Effects of ZnO NPs on antioxidant enzymes and other biochemical indicators of cotton seedlings under salt stress. (**A**) superoxide dismutase activity (SOD); (**B**) peroxidase activity (POD); (**C**) catalase activity (CAT); (**D**) MDA content; (**E**) H_2_O_2_ content; (**F**) O_2_^−^ content; (**G**) proline content; (**H**) protein content; (**I**) amino acid content. Control (CK), CK treated with 100 mg/L ZnO NPs (CZ), CK treated with 150 mM NaCl (NK), and CZ treated with 100 mg/L ZnO NPs (NZ). The data given are the averages of three replicates, with the standard deviation (SD) shown by the error bars. Different letters above or below the error bars show the differences at *p* < 0.05.

To find the function of ZnO NPs on the antioxidative system in cotton seedlings, we determined the activities of antioxidant enzymes in cotton (Figure 4). Six days of 150 mM NaCl treatment greatly decreased the activities of antioxidant enzymes (SOD and CAT) (Figure 4A–C) and increased the contents of MDA, H_2_O_2_, and O_2_^−^ (Figure 4D–F) associated with the control. In the salt-stress-treated cotton seedlings, spraying 100 mg/L ZnO NPs significantly induced the SOD, POD, and CAT activities by 45.40%, 39.59%, and 22.77% in the cotton leaves than the salt-stress treatment alone, respectively (Figure 4A–C), and notably reduced the MDA, H_2_O_2_, and O_2_^−^ by 16.96%, 11.90%, and 11.38%, respectively (Figure 4D–F). Our findings indicate that the spraying of ZnO NPs on cotton plants exposed to 150 mM NaCl might alleviate redox toxicity by inducing the activities of antioxidant enzymes and reducing the ROS. In addition, the contents of protein and amino acid also increased significantly after spraying ZnO NPs (Figure 4H,I), and the contents of proline increased slightly, but there was no significant difference (Figure 4G).

### 3.5. Analysis of the Effects of ZnO NPs on Gene Expression in the Salt-Responsive Transcriptome of Cotton

To gain insight into the function of exogenously sprayed ZnO NPs to alleviate salt stress in cotton, we conducted RNA-Seq analysis to screen for differentially expressed genes (DEGs). A total of four treatments were set up in this study, namely CK, ZnO NPs (100 mg/L), CK + NaCl, and ZnO NPs (100 mg/L) + NaCl, which were named CK, ZnO, CK + NaCl, and ZnO + NaCl, respectively, during sequencing analysis, and each treatment was subjected to three independent biological replications, with the sampling time point of each treatment being 24 h after the salt-stress treatments, and the sampling site being the third true leaf of the cotton seedling (from the bottom up).

High-quality clean bases of 20.71, 20.87, 19.78, and 21.60 Gb were obtained for CK, ZnO, CK + NaCl, and ZnO + NaCl by RNA-seq, respectively, and the error rates of the sequencing data ranged from 0.01% for all treatments, above 98% for Q20 and 96% for Q30, and between 43.78 and 44.62% for the GC content (Table S1), indicating that the quality of the sequencing data was good. After filtering, the clean reads were 94.78% to 96.24% when associated with the reference genome of upland cotton (Table S2). In addition, the current correlation coefficients of the three replicates of the four materials were all around 0.98 (Figure S2), indicating that the reproducibility among the treated samples is good and the subsequent test data are reliable.

There were a total number of 29,863 DEGs found between ZnO + NaCl and CK + NaCl (4653), CK + NaCl and CK (10,035), ZnO and CK (5786), and ZnO + NaCl and ZnO (9389) after screening all the materials (Table S3). The number of DEGs in the CK + NaCl vs. CK (10,035) was more than twice that in the ZnO + NaCl vs. CK + NaCl (4653), and the number of DEGs in the ZnO + NaCl vs. CK + NaCl (4653) was a bit lower than that in the ZnO vs. CK (5786), which may indicate that the exogenous spraying of ZnO NPs can mitigate the toxic effects of a high-salt environment on cotton seedlings.

**Figure 5:**
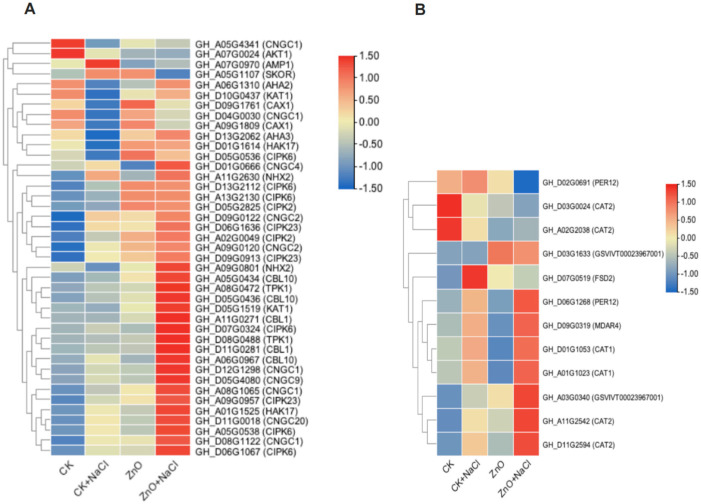
Heat maps of differentially expressed genes after spraying ZnO NPs on cotton seedlings under salt stress. (**A**) Differential expression of genes related to ion stress; (**B**) differential expression of genes related to osmotic stress.

Afterward, Gene Ontology (GO) and Kyoto Encyclopedia of Genes and Genomes (KEGG) enrichment analyses of up-regulated and down-regulated genes were performed. By GO enrichment analysis of down-regulated genes in the comparison of ZnO + NaCl and CK + NaCl, it was found that a total of 70 GO terms were significantly enriched. And ion stress-related GO terms, including “cell wall organization or biogenesis”, “cell wall organization”, and “cell wall biogenesis” were significantly enriched (Figure S3B). It is noteworthy that the “sulfur compound biosynthetic process” pathway was significantly down-regulated in GO enrichment, which is consistent with the reduction of sulfur content by spraying ZnO NPs on cotton seedlings under salt stress in the previous elemental results. Additionally, by KEGG enrichment analysis of upregulated genes in comparison to ZnO + NaCl vs. CK + NaCl, it was found that a total of 12 pathways were significantly enriched. Among them, the second richest was the MAPK signaling pathway-plant pathway, with 47 genes annotated, which was often found to be significantly altered in response to salt stress. “Phenylpropanoid biosynthesis”, “Alanine, aspartate and glutamate metabolism”, and “Amino sugar and nucleotide sugar metabolism” were also common up-regulated pathways in response to salt stress (Figure S3C). In addition, the analysis also found that many genes related to sodium-potassium ion transport and calcium ion signal transduction were significantly up- or down-regulated (Figure 5A), as were antioxidant-related genes (Figure 5B).

## 4. Discussion

Studies have shown that salt stress greatly reduces plant growth traits by interfering with ion homeostasis and osmotic regulation in plants [33]. ZnO NPs can improve crop tolerance and plant growth performance under various abiotic stresses, such as salt stress. In this study, the foliar spraying of ZnO NPs on cotton leaves was found to make the cotton seedlings show better phenotypes, increased the chlorophyll content and antioxidant enzyme activities, and decreased the Na^+^/K^+^ ratio, MDA, H_2_O_2_, and O_2_^−^, thus maintaining ion homeostasis and reducing the oxidative damage of cotton under salt stress. The results of previous studies have shown that the foliar spraying of ZnO NPs significantly increased the aboveground biomass, leaf area, photosynthetic characteristics, protein content, and antioxidant enzyme activities of tomato plants, which play an important role in alleviating salinity toxicity in tomato plants [27]. The foliar spraying of ZnO NPs on barley plants was also found to reduce Na^+^ uptake, increase Zn^2+^ uptake, inhibit the production of reactive oxygen species, and alleviate growth inhibition in barley under salt stress [29], which is consistent with the results of this study. When the normal growth and development of a plant changes, the plant’s photosystem II (PS II) will be changed, for example, the physiological or pathological changes caused by salt stress and other adversity stresses can lead to the impaired function of PS II [34]. Therefore, when environmental conditions change, changes in the chlorophyll fluorescence of plants can reflect, to some extent, whether the environment affects the plant. In this study, the foliar spraying of ZnO NPs significantly improved the chlorophyll fluorescence parameters, such as the Fv/FM and ABS/CS of cotton seedlings under salt stress, indicating that ZnO NPs could reduce the damage of salt stress on the cotton’s photosystem II.

Under salt stress, a high concentration of Na^+^ competes with K^+^ uptake by plants, resulting in a leakage of K^+^ through the outward-rectified K^+^ channels. This reduces the K^+^/Na^+^ ratio, which causes plant Na^+^ toxicity, results in biochemical metabolism disorders in the plant body, and affects the normal growth and development, which in turn leads to the inhibition of plant growth or even death. Therefore, the stability of the plant Na^+^/K^+^ ratio is a key indicator of the strength of the plant’s salt tolerance [35]. According to the results of the elemental measurements, the sodium content was significantly increased in the leaves of the plants under salt stress compared to those of the control, whereas the sodium content was significantly decreased and the potassium content was slightly increased after spraying with ZnO NPs, but there was no significant difference. Through in-depth analysis by RN-Seq assay, we found that the expressions of relevant genes regulating Na^+^ and K^+^ changed significantly after spraying ZnO NPs on cotton seedlings under salt stress. The *CNGC* family of genes, including *CNGC1*, *CNGC2*, *CNGC4*, *CNGC9*, *CNGC13*, and *CNGC20*, were significantly up-regulated (Figure 5A), whereas the plant *CNGC* gene family plays a role in multiple processes of plant growth, development, and resistance to adversity stress, and it negatively regulates the influx of Na^+^ and mitigates the toxic effects of excess Na^+^ on plants under salt stress [36]. Plants can reduce the toxicity of Na^+^ to plants through two pathways: the increased efflux of Na^+^ and transport to the vesicles. In this study, we found that *AMP1* was significantly down-regulated and *AHA3* was significantly up-regulated after spraying ZnO NPs (Figure 5A). Previous studies have shown that the deficiency of *AMP1* promotes the efflux of Na^+^, and *AMP1* negatively regulates the expression of the *AHA3* gene, which mediates the signaling process for resistance to salt stress and reduces Na^+^ levels [37], which is consistent with the results of this study. The vesicular membrane Na^+^/H^+^ reverse transporter protein (NHX) compartmentalizes Na^+^ from the cytoplasm to the vesicle, eliminating Na^+^ toxicity while promoting the high-affinity uptake of K^+^ [38]. Previous studies have found that overexpression of the wheat *TaNHX2* gene in cotton significantly enhanced salt and drought tolerance in transgenic cotton [39]. In this study, *NHX2* was significantly up-regulated after spraying ZnO NPs compared to the control plants under salt stress (Figure 5A), suggesting that more Na^+^ was compartmentalized to the vesicles, which mitigated the toxic effects on the cotton seedlings. In addition, *HAK17* was dramatically up-regulated and *SKOR* was distinctly down-regulated after spraying ZnO NPs (Figure 5A). *HAK* is a high-affinity K^+^ transporter protein that enhances salt tolerance in plants by maintaining plant Na^+^/K^+^ homeostasis and attenuating Na^+^ toxicity [40]. *SKOR*, as an external rectifier K^+^ channel protein, is mainly involved in K^+^ loading from the column cell to the xylem [41]. Therefore, ZnO NPs could elevate K^+^ uptake and reduce K^+^ loss in the cotton seedlings under salt stress.

**Figure 6:**
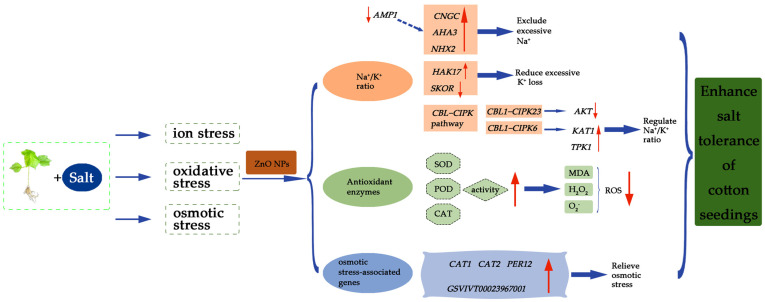
The mechanism model of ZnO NPs alleviating salt stress in cotton seedlings.

The *CBL-CIPK* pathway is an important mechanism for plant resistance to different external stresses that trigger Ca^2+^ signaling events, such as salt stress [42]. Calcium sensors *CBL1* and *CBL9* synergistically regulate *CIPK23*, which in turn targets and activates the potassium channel *AKT1*, thereby enhancing the absorption of K^+^ by plants [43]. Previous studies have found that under drought stress, *AtCBL1* expression was induced in cell lines overexpressing *ghcipk6*, along with an increase in the expression of the inward K^+^ channels *AtKAT1*, and *AtTPK1* [44]. In this experiment, *CIPK6*, *CBL1*, *KAT1*, and *TPK1* were all significantly up-regulated after spraying ZnO NPs, which is consistent with the results of previous studies, indicating that the plant alleviated salt stress damage through the *CBL1-CIPK6* pathway. *CIPK2* is required for salt tolerance and may act as a salt-triggered signaling sensor to primarily regulate K^+^ transport in salt stress signaling. The overexpression of *HbCIPK2* in *Arabidopsis* could maintain Na^+^/K^+^ homeostasis and protect root cells from death to enhance salt tolerance [45]. In this experiment, genes of the *CIPK2* family were significantly up-regulated (Figure 5A), regulating K^+^ transport to maintain Na^+^/K^+^ homeostasis. In addition, *CAX* protein is involved in various abiotic stress pathways and also plays a regulatory role in plants under salt stress. For example, increasing the expression of soybean *CAX (GmCAX1)* can increase its salt tolerance [46]. Changes in *CAX1* transporters can affect cellular Ca^2+^ flux and mediate salt-stress-related responses [47]. In this study, *CAX1* was also significantly up-regulated after spraying ZnO NPs (Figure 5A), and Ca^2+^ was significantly increased after spraying ZnO NPs on cotton seedlings under salt stress (Figure 3G), suggesting that ZnO NPs were able to relieve ion stress by maintaining intracellular Na^+^/K^+^ stabilization in the plants under salt stress and enhance the salt tolerance of the cotton seedlings (Figure 6).

Over-accumulated ROS (including MDA, H_2_O_2_, and O_2_^−^), could damage the protein, DNA, membrane, and other biomolecules in plants [5,48]. The production of ROS can be scavenged by antioxidative defense systems, such as superoxide dismutase (SOD), catalase (CAT), and peroxidase (POD) [48]. After the foliar spraying of ZnO NPs, we discovered that the MDA, H_2_O_2_, and O_2_^−^ contents were notably reduced, and the SOD, POD, and CAT activities were improved, mitigating the high-salinity stress in our research (Figure 4F). These results are similar to the mechanism of arbuscular mycorrhizal fungi (AMF) vaccination in alleviating oxidative stress of maize plants under salt stress in the study of Ahmed M. El-Sawah et al. [49]. Moreover, plenty of osmotic stress-associated genes (such as *CAT1*, *CAT2*, *MDAR4*, and *GSVIVT00023967001*) were significantly induced after ZnO NPs spraying under the 150 mM NaCl treatment (Figure 5B). This suggests that ZnO NPs regulate antioxidant enzymes and alleviate salt-induced oxidative stress and osmotic stress (Figure 6).

Because zinc is a heavy metal element, its content exceeding a certain value will have a toxic effect on plants, and excessive zinc may also cause environmental pollution. In addition, it was found that the P content in cotton leaves was significantly reduced after spraying ZnO NPs. Therefore, further studies are needed on how to better apply ZnO NPs to alleviate various crop stresses while maintaining the stability of P/Zn. Therefore, although ZnO NPs have good development potential in alleviating abiotic stress, such as salt stress in cotton, further evaluation of its impact on the environment and its application in production are still needed.

## 5. Conclusions

To summarize, this study reveals that the foliar spraying of ZnO NPs could effectively alleviate the inhibition effect of salt stress on cotton seedlings, and the concentration of 100 mg/L had the best effect. ZnO NPs improved the phenotypic characteristics, increased the chlorophyll content and chlorophyll fluorescence parameters, such as F_v_/F_M_, and enhanced the ROS scavenging ability of cotton seedlings under salt stress. It is worth noting that ZnO NPs eliminated excess Na^+^ and reduced the loss of K^+^ through ion stress-related genes, thus stabilizing the Na^+^/K^+^ ratio under high salt stress in cotton and enhancing the salt tolerance of the cotton seedlings. Consequently, this research reveals the possible potential mechanism of ZnO NPs in alleviating salt stress in cotton, provides a new method for alleviating salt stress in cotton, and also demonstrates the great potential of nanomaterials for alleviating crop abiotic stress.

## Data Availability

Data will be made available upon request.

## Supplementary Materials

The following supporting information can be downloaded at: https://www.mdpi.com/article/10.3390/life14050595/s1, Figure S1: ZnO NPs alleviate salt stress in cotton. Shoot dry weights (A), root dry weights (B), and stem diameters (C) of cotton seedlings under salt stress subjected to 0, 50, 100, 150, and 200 mg/L of ZnO NPs; Figure S2: Plot of results of principal component analysis; Figure S3: Significant up- and down-regulated pathway map of GO and KEGG enrichment; Table S1: Summary of transcriptome sequencing data; Table S2: Summary of mapped reads in transcriptome sequencing data; Table S3: Statistics of differentially expressed genes in the transcriptome.
