# Supplementary material for: Zinc Oxide Nanoparticles Alleviate Salt Stress in Cotton (Gossypium hirsutum L.) by Adjusting Na+/K+ Ratio and Antioxidative Ability
Source: Life (Basel). 2024 May 7;14(5):595. doi: 10.3390/life14050595 (PMC11121869; doi:10.3390/life14050595)
Supplement: Supplementary file 1 [file life-14-00595-s001.zip › life-2983713-supplementary.pdf]

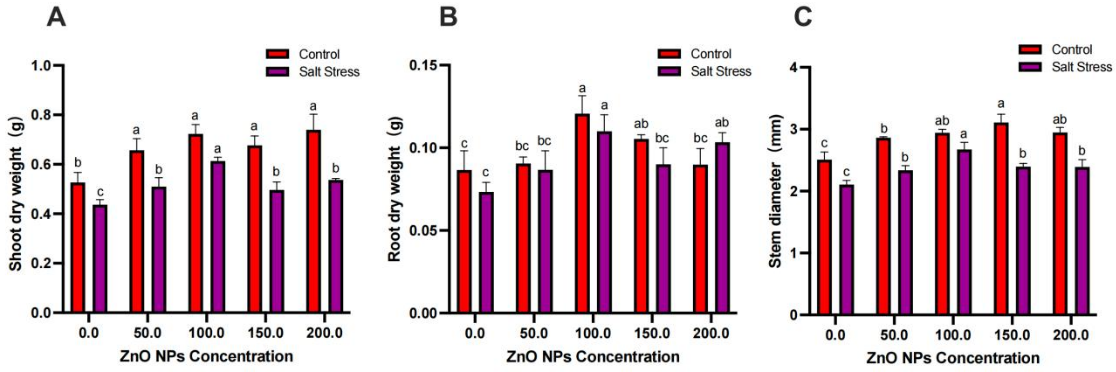


**Fig S1** ZnO NPs alleviate salt stress in cotton. Shoot dry weight (A), root dry weight (B), stem diameter (C) of cotton seedlings subjected to 0, 50, 100, 150 and 200 mg/L of ZnO NPs under salt stress were measured, respectively. The tests were repeated five times. The data given are the averages of three replicates, with the standard deviation (SD) shown by the error bars. Different letters above or below error bars show the differences at $p < 0.05$.

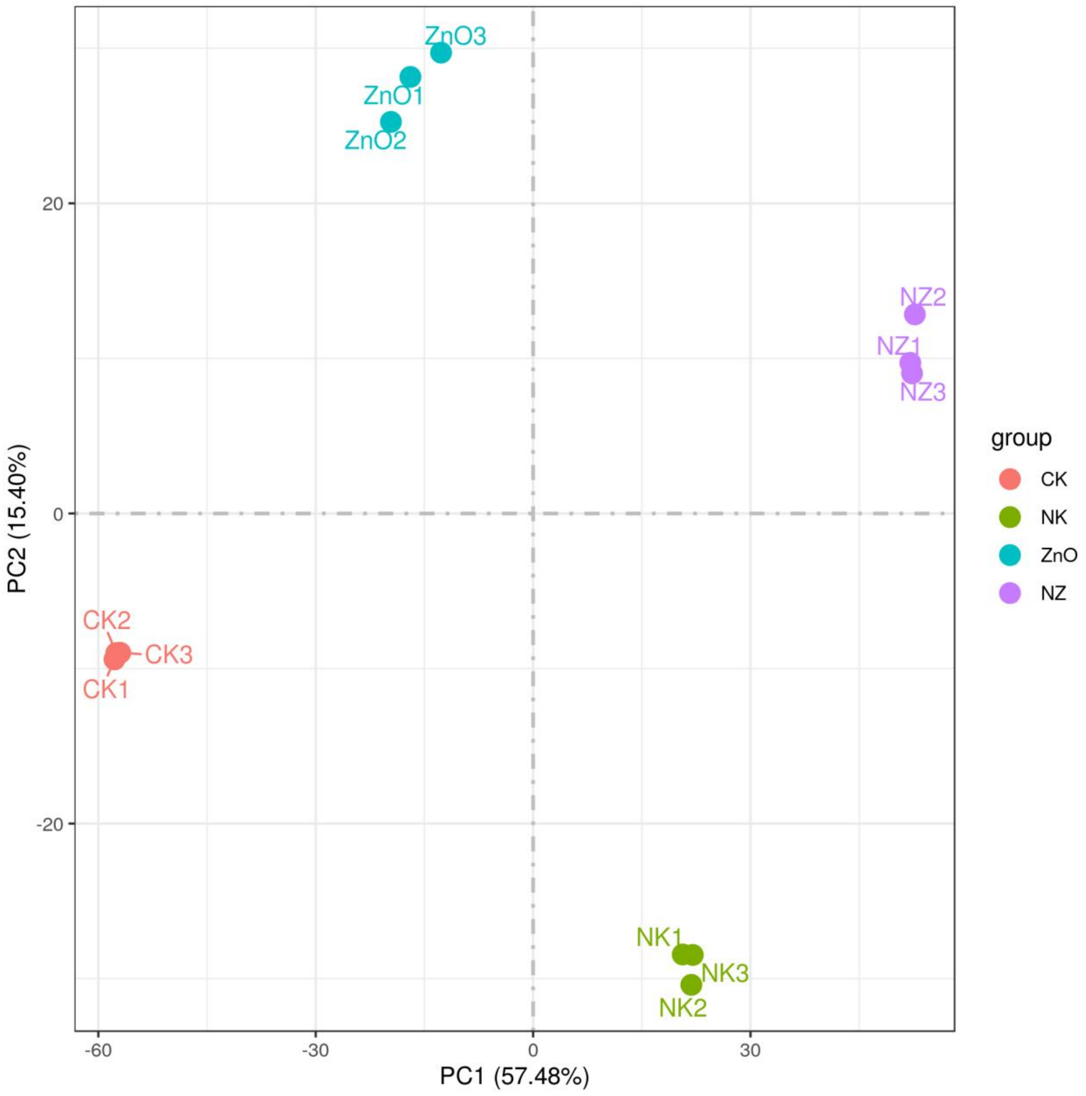


**Fig S2** Plot of results of principal component analysis

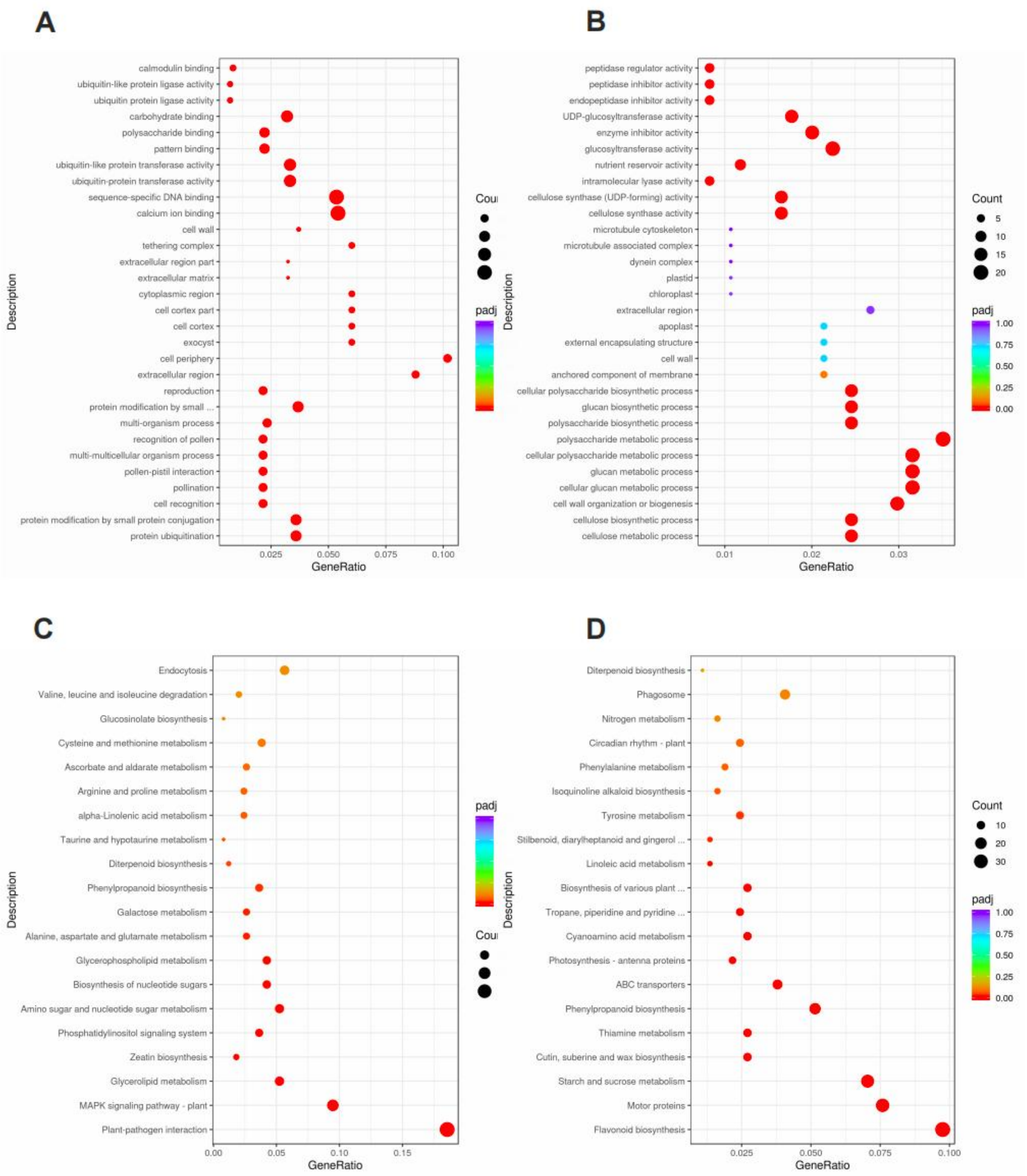


**Fig S3** Significant up and down-regulated pathway map of GO and KEGG enrichment (NZvsNK). GO enrichment up-regulated gene (A), GO enrichment down-regulated gene (B), KEGG enrichment up-regulated gene (C), KEGG enrichment down-regulated gene (D). NK and NZ represent CK-NaCl, and ZnO (100 mg/L)-NaCl, respectively.

**Table S1** Summary of transcriptome sequencing data

| Library | Raw reads (bp) | Clean reads (bp) | Clean reads (%) | Clean bases (Gb) | Error rate (%) | Q20 (%) | Q30 (%) | GC content (%) |
|---|---|---|---|---|---|---|---|---|
| CK1 | 46092874 | 44364618 | 96.25 | 6.65 | 0.01 | 98.93 | 96.89 | 44.47 |
| CK2 | 47938760 | 45975326 | 95.90 | 6.9 | 0.01 | 98.88 | 96.77 | 43.94 |
| CK3 | 50042226 | 47752296 | 95.42 | 7.16 | 0.01 | 98.94 | 96.87 | 44.11 |
| ZnO1 | 46821004 | 44998716 | 96.11 | 6.75 | 0.01 | 98.87 | 96.65 | 43.83 |
| ZnO2 | 50721768 | 48775556 | 96.16 | 7.32 | 0.01 | 98.92 | 96.82 | 44.62 |
| ZnO3 | 47000548 | 45324198 | 96.43 | 6.8 | 0.01 | 98.95 | 96.96 | 44.43 |
| NK1 | 47461958 | 45727572 | 96.35 | 6.86 | 0.01 | 98.93 | 96.83 | 43.93 |
| NK2 | 40210746 | 38179350 | 94.95 | 5.73 | 0.01 | 98.83 | 96.67 | 43.8 |
| NK3 | 49430364 | 47908868 | 96.92 | 7.19 | 0.01 | 98.89 | 96.77 | 44.08 |
| NZ1 | 48930656 | 46885310 | 95.82 | 7.03 | 0.01 | 98.86 | 96.7 | 43.97 |
| NZ2 | 51571142 | 48458764 | 93.96 | 7.27 | 0.01 | 98.91 | 96.8 | 43.78 |
| NZ3 | 50129572 | 48675432 | 97.10 | 7.3 | 0.01 | 98.86 | 96.65 | 43.93 |

Note: CK, ZnO, NK and NZ represent CK, ZnO (100 mg/L), CK-NaCl, and ZnO (100 mg/L)-NaCl, respectively, and 1-3 represent 3 independent biological replicates, respectively.

**Table S2** Summary of mapped reads in transcriptome sequencing data

| Sample | Total reads | Mapped reads | Mapped rate (%) | Uniquely mapped | Multiple mapped |
|---|---|---|---|---|---|
| CK1 | 44364618 | 42068483 | 94.82 | 39999300 | 2069183 |
| CK2 | 45975326 | 44020519 | 95.75 | 41872141 | 2148378 |
| CK3 | 47752296 | 45351419 | 94.97 | 43033396 | 2318023 |
| ZnO1 | 44998716 | 42967376 | 95.49 | 41025161 | 1942215 |
| ZnO2 | 48775556 | 46444358 | 95.22 | 44327255 | 2117103 |
| ZnO3 | 45324198 | 43067190 | 95.02 | 41157771 | 1909419 |
| NK1 | 45727572 | 43740558 | 95.65 | 41595994 | 2144564 |
| NK2 | 38179350 | 36323638 | 95.14 | 34503821 | 1819817 |
| NK3 | 47908868 | 45406572 | 94.78 | 43297677 | 2108895 |
| NZ1 | 46885310 | 45122046 | 96.24 | 43144957 | 1977089 |
| NZ2 | 48458764 | 46194735 | 95.33 | 44022569 | 2172166 |
| NZ3 | 48675432 | 46449633 | 95.43 | 44449272 | 2000361 |

Note: CK, ZnO, NK and NZ represent CK, ZnO (100 mg/L), CK-NaCl, and ZnO (100 mg/L)-NaCl, respectively, and 1-3 represent 3 independent biological replicates, respectively.

**Table S3** Statistics of differentially expressed genes in the transcriptome

| Compare | No. of DEGs | No. of upregulated genes | No. of downregulated genes |
|---|---|---|---|
| NZvsNK | 4653 | 2985 | 1668 |
| NKvsCK | 10035 | 4127 | 5908 |
| ZnOvsCK | 5786 | 3024 | 2762 |
| NZvsZnO | 9389 | 3999 | 5390 |

Note: CK, ZnO, NK and NZ represent CK, ZnO (100 mg/L), CK-NaCl, and ZnO (100 mg/L)-NaCl, respectively.
